# ^89^Zr-girentuximab PET/CT Enables Noninvasive Assessment of Indeterminate Renal Masses and Metastatic Clear-Cell Renal Cell Carcinoma

**DOI:** 10.3390/pharmaceutics18020258

**Published:** 2026-02-19

**Authors:** Yihan Cao, Jonathan Kim, Justin Talluto, Taylor McVeigh, Michael L. Blute, Douglas M. Dahl, Keyan Salari, Pedram Heidari, Shadi A. Esfahani

**Affiliations:** 1 Division of Nuclear Medicine and Molecular Imaging, Department of Radiology, Massachusetts General Hospital, Harvard Medical School, Boston, MA 02114, USA; ycao@mgh.harvard.edu (Y.C.); jkim215@mgh.harvard.edu (J.K.);; 2 Department of Urology, Massachusetts General Hospital, Harvard Medical School, Boston, MA 02114, USA

**Keywords:** ^89^Zr-girentuximab, positron emission tomography/computed tomography, clear-cell renal cell carcinoma, tumor thrombus, oncocytoma

## Abstract

**Background:** Indeterminate renal masses (IRMs) frequently require biopsy for characterization and often lead to unnecessary surgical interventions. ^89^Zr-girentuximab is a positron emission tomography (PET) radiopharmaceutical targeting carbonic anhydrase IX, a biomarker overexpressed in clear-cell renal cell carcinoma (ccRCC). This real-world experience demonstrates the impact of ^89^Zr-girentuximab PET on the clinical management of patients with IRM and its role in differentiating primary and metastatic ccRCC from other etiologies. **Methods:** This prospective single-center study, part of an expanded access program (NCT06090331), investigated patients with IRM on conventional imaging who underwent ^89^Zr-girentuximab PET/computed tomography (PET/CT). Qualitative and quantitative PET/CT features of each lesion were assessed. Pathologic or clinical diagnosis was determined for all lesions. Referring physicians were surveyed to evaluate the impact of PET on patient management. **Results:** Seven male patients (age range, 57–78 years) were included; four had ccRCC (including two with metastatic disease) and three had oncocytoma (including one with Birt-Hogg-Dubé syndrome). Across all 32 lesions identified, ^89^Zr-girentuximab PET/CT accurately characterized each lesion based on pathologic or clinical diagnosis. ^89^Zr-girentuximab PET/CT identified ccRCC tumor thrombi in the inferior vena cava and renal vein branches (SUV_max_ 12.0–13.0), a perinephric deposit (SUV_max_ 36.4), and intramuscular (SUV_max_ 103.0), pulmonary (SUV_max_ 4.0–10.5), and osseous (SUV_max_ 10.2) metastases. ^89^Zr-girentuximab PET/CT enabled the diagnosis of oncocytomatosis in one patient and detected a renal lesion with positive uptake that was occult on MRI. According to referring physicians, ^89^Zr-girentuximab PET/CT changed clinical management in six of seven patients and improved patient care in all cases. **Conclusions:** ^89^Zr-girentuximab PET/CT provides a noninvasive tool for characterizing indeterminate renal masses and metastatic ccRCC and may improve clinical problem-solving in complex scenarios.

## 1. Introduction

Indeterminate renal masses (IRMs) are a common clinical scenario. Approximately 14% of patients undergoing computed tomography (CT) have an incidental renal mass, of which 13% ultimately remain indeterminate [1]. While some lesions, such as clear-cell renal cell carcinoma (ccRCC), exhibit malignant biological behavior, a substantial proportion are benign or indolent and may not require treatment [2]. Studies show that approximately 20–30% of small renal masses (≤4 cm) are benign, and among those identified as RCC, 70–80% are low-grade tumors with limited expected malignant potential [3]. On the other hand, conventional imaging and even needle biopsy have significant limitations in differentiating these lesions, leading to long-term frequent follow-up imaging or overtreatment with surgery. Up to 30% of resected small renal masses are ultimately found to be benign [4,5]. Such unnecessary surgeries carry risks of complications and renal function loss [2].

^89^Zr-girentuximab is a radiopharmaceutical for positron emission tomography (PET) imaging that targets carbonic anhydrase IX (CAIX), a cell-surface antigen expressed in approximately 95% of clear-cell renal cell carcinomas and rarely in normal renal tissue or benign renal lesions [6,7,8]. Recently published phase III ZIRCON trial demonstrated its high diagnostic accuracy for detection and characterization of ccRCC in IRMs, with a sensitivity of 85.5% and specificity of 87.0% [9], suggesting its potential to alter the current diagnostic paradigm [10]. In addition, ^89^Zr-girentuximab positron emission tomography/computed tomography (PET/CT) may support clinical decision-making in patients with confirmed ccRCC by characterizing indeterminate lesions and evaluating disease extent and metastasis [11]. Nevertheless, real-world data on its impact on clinical management remains limited.

In this study, we report our single-center experience with ^89^Zr-girentuximab PET/CT, highlighting its role in distinguishing primary and metastatic ccRCC from benign lesions and in aiding clinical problem-solving in complex cases.

## 2. Materials and Methods

### 2.1. Patients

In this prospective single-center study as part of an expanded access program (EAP) (NCT06090331), patients aged ≥18 years with renal mass(es) identified on conventional imaging such as CT or magnetic resonance imaging (MRI) were enrolled between October 2024 and May 2025. Patients were referred to the study by treating physicians in the Department of Urology at Massachusetts General Hospital. Key exclusion criteria included renal masses known to be metastases from other primary malignancies, active non-renal malignancies requiring treatment, receipt of radiotherapy or immunotherapy within 4 weeks prior to planned administration of ^89^Zr-girentuximab, estimated glomerular filtration rate ≤ 40 mL/min/1.73 m^2^, or serious non-malignant conditions (e.g., psychiatric, infectious, autoimmune, or metabolic disorders) that could compromise patient safety or study compliance. The complete eligibility criteria are available on ClinicalTrials.gov [12]. Written informed consent was obtained from all participants.

Patients’ demographic and clinical characteristics were recorded, including the indication for ^89^Zr-girentuximab PET/CT and laboratory findings such as renal function, complete blood count, liver function tests, and coagulation panel obtained within 30 days of ^89^Zr-girentuximab injection. Pathologic diagnosis from biopsy or surgical excision after the PET imaging was used when available. For lesions without available pathology, a clinical diagnosis was established based on the integrated assessment of ^89^Zr-girentuximab PET/CT findings, conventional imaging, and clinical context. End of treatment (EOT) was defined as 10 ± 2 days post-injection. For the safety follow-up period, patients were contacted by phone at EOT and once more 30 ± 10 days from EOT to assess for adverse events. Referring physicians were surveyed to assess the impact of PET on patient management at least 90 days after EOT. Participants were reimbursed for travel for this study.

### 2.2. ^89^Zr-girentuximab Preparation, Handling, and Administration

^89^Zr-girentuximab was provided by Telix Pharmaceuticals and manufactured in accordance with current Good Manufacturing Practice requirements as previously described [9]. Briefly, girentuximab, a chimeric monoclonal antibody specific for CAIX, was radiolabeled with the positron-emitting radiometal zirconium-89 via an N-succinyl-desferrioxamine-tetrafluorophenyl ester (N-Suc-DFO-TFP) bifunctional chelator, which was covalently coupled to lysine residues on the antibody. The final drug product was formulated as a solution in a syringe at a decay-corrected activity of 37 MBq (±10%, 10 mg girentuximab) for the time of administration without further modification.

Each syringe was assayed using a dose calibrator before and after administration to determine the injected radioactive activity. Individual patient doses were prepared centrally by a radiopharmaceutical contract manufacturer and were released with a batch-specific certificate of analysis, confirming that all preestablished release specifications were met. At our institution, an academic medical center operating under a radioactive materials license for medical use, the investigational product was received, stored, and handled by qualified radiopharmacists in accordance with institutional and regulatory standards.

Patients received a single intravenous dose of ^89^Zr-girentuximab (37 MBq ± 10%) administered by a certified nuclear medicine technologist under the supervision of a nuclear medicine physician investigator over a minimum of three minutes, followed by a flush of at least 15 mL of normal saline. A 0.22-µm filter was placed between the syringe and injection line during radiopharmaceutical administration.

### 2.3. PET/CT Acquisition and Interpretation

Patients underwent abdominal or whole-body PET/CT imaging 5 ± 2 days after injection on the Biograph Vision 600 PET/CT scanner (Siemens Healthineers, Knoxville, TN, USA). The imaging field of view was determined based on clinical indication. Abdominal PET was acquired using a single bed position with an acquisition time of 10–20 min. Whole-body PET involved 7–8 bed positions with an acquisition time of 5 min per bed position. PET images were reconstructed using a time-of-flight algorithm, and the reconstructed images were used for analysis. A low-dose CT scan was performed for attenuation correction. The detailed PET/CT acquisition protocol is provided in Supplementary Table S1.

Image interpretation was conducted using the Syngo Via RT Image Suite VB40 (Siemens Healthineers, Forchheim, Germany) by a board-certified nuclear radiologist investigator at our institution with over ten years of experience in PET/CT interpretation. PET positivity was determined qualitatively by visual assessment: lesions were considered positive if tracer uptake was higher than that of adjacent normal tissue, and negative if uptake was similar to or lower than adjacent normal tissue background [9]. For quantitative analysis, the maximum standardized uptake value (SUV_max_) of each lesion was measured using manually drawn volumes of interest. The mean standardized uptake value (SUV_mean_) of adjacent normal tissue within the same organ was recorded. Additionally, SUV_mean_ were measured in the right hepatic lobe and the abdominal aorta to represent liver and blood pool background activity, respectively. The maximum diameter of each lesion was determined using the most recent anatomic imaging performed within 90 days of the ^89^Zr-girentuximab PET/CT. For pulmonary nodules, only lesions ≥ 8 mm (at least twice the PET scanner spatial resolution) were included for measurement.

### 2.4. Statistical Analysis

Statistical analysis was performed using R (version 4.4.2; © R Foundation for Statistical Computing, Vienna, Austria). Continuous variables were summarized as medians with ranges. Quantitative PET metrics were analyzed, including lesion SUV_max_, lesion-to-background uptake ratio (lesion SUV_max_/adjacent normal tissue SUV_mean_), lesion-to-liver uptake ratio (lesion SUV_max_/liver SUV_mean_), and lesion-to-blood pool uptake ratio (lesion SUV_max_/blood pool SUV_mean_). Comparisons were made between ccRCC and non-ccRCC lesions based on pathologic or clinical diagnosis, and between primary and metastatic ccRCC lesions. Group differences were assessed using the Mann–Whitney U test. A *p*-value < 0.05 was considered statistically significant.

## 3. Results

### 3.1. Patient Characteristics

Seven non-Hispanic White male patients (age range, 57–78 years; median, 67 years) were screened, all of whom met eligibility criteria and were enrolled in this study. Among them, four had pathology-confirmed ccRCC, including two with metastatic ccRCC, and three had pathology-confirmed oncocytoma, including one with Birt–Hogg–Dubé syndrome. Five patients presented with incidental renal masses, one with suspected recurrent ccRCC following a partial nephrectomy, and one with an enlarging renal mass identified during surveillance of Bosniak IIF renal cysts. Detailed patient characteristics are summarized in Table 1.

The median body weight was 82.6 kg (range, 76.2–123.8 kg), and the median body mass index was 27.9 kg/m^2^ (range, 24.8–39.2 kg/m^2^). The median estimated glomerular filtration rate at the time of ^89^Zr-girentuximab administration was 61 mL/min/1.73 m^2^ (range, 41–90 mL/min/1.73 m^2^). Four patients underwent abdominal PET/CT and three underwent whole-body PET/CT, with acquisition times of 10–20 min and 35–40 min, respectively. All patients completed the safety follow-up period 21 to 32 days after EOT. All referring physicians completed their surveys 90–100 days after EOT. One patient reported hematuria one day after the scan, which was determined to be related to the recent biopsy and not related to the study drug. No related adverse events were reported.

### 3.2. Lesion Features

A total of 32 lesions (size range, 8–131 mm; median, 18.5 mm) were identified on ^89^Zr-girentuximab PET/CT, of which five were pathology-confirmed ccRCC (including one psoas muscle metastasis), and three were biopsy-proven oncocytomas. The remaining 24 lesions were diagnosed clinically based on ^89^Zr-girentuximab PET/CT uptake pattern, conventional imaging characteristics, and clinical context, with 13 ccRCC and 11 non-ccRCC lesions. No false positive or false negative lesions by ^89^Zr-girentuximab PET/CT were detected. CAIX immunohistochemistry was available for four of the eight pathology-confirmed lesions, three of which were positive in ccRCC and one negative in oncocytoma. The detailed lesion characteristics are summarized in Table 1.

**Figure 1:**
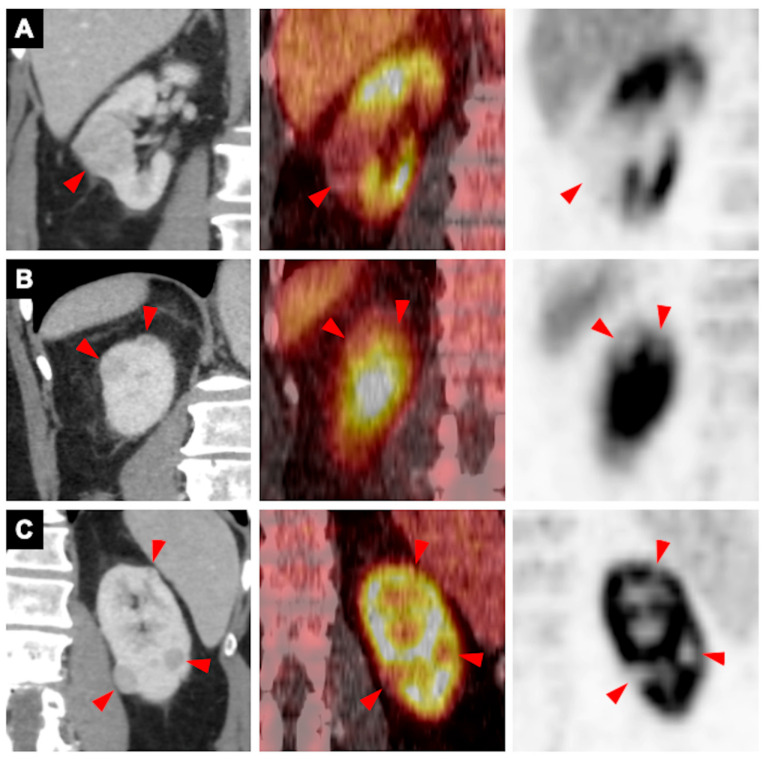
A 76-year-old man with Birt–Hogg–Dubé syndrome presented with multiple indeterminate renal masses. Coronal portal venous phase CT images (**left**), ^89^Zr-girentuximab PET/CT fused images (**middle**), and PET images (**right**) demonstrate multiple solid renal masses, with three in the right kidney (triangles in (**A**,**B**)) and three in the left kidney (triangles in (**C**)). All lesions show lower ^89^Zr-girentuximab uptake compared with the normal renal parenchyma. Biopsy of the largest right renal lesion (triangles in (**A**)) revealed an oncocytoma. Based on the biopsy and PET/CT findings, a diagnosis of renal oncocytomatosis was established, and the patient was managed with active surveillance.

**Figure 2:**
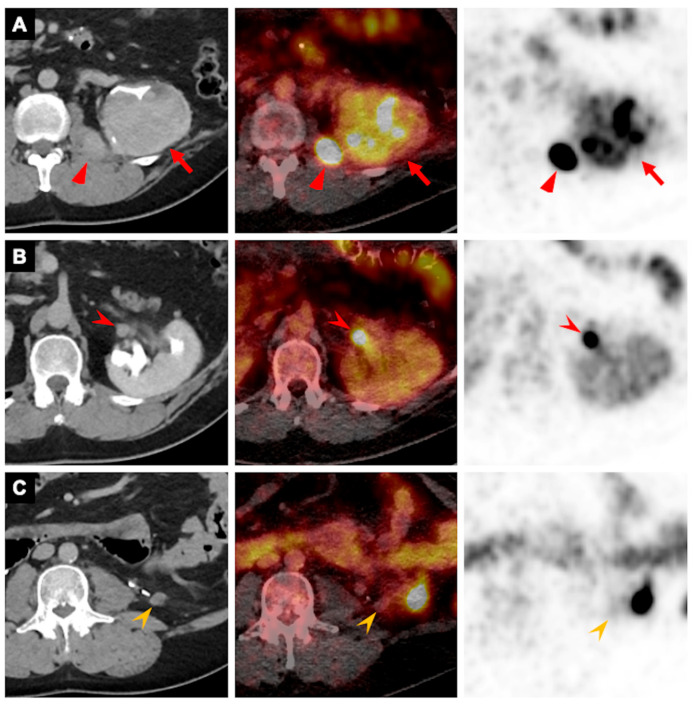
A 57-year-old man with a history of clear-cell renal cell carcinoma status post left partial nephrectomy ten years earlier presented with gross hematuria and was found to have suspected local recurrence. Axial portal venous phase CT images (**left**), ^89^Zr-girentuximab PET/CT fused images (**middle**), and PET images (**right**) demonstrate an enhancing left renal mass with heterogeneously intense uptake (arrows in (**A**)) and an enhancing, intensely avid nodule in the left psoas muscle (triangles in (**A**)). A perinephric soft-tissue nodule is seen anteriorly with intense uptake (arrowheads in (**B**)), suspicious for tumor deposit. An additional inferior nodule shows no uptake (arrowheads in (**C**)) and is unchanged compared with the postoperative CT obtained nine years earlier, consistent with benign postoperative changes. The patient subsequently underwent left radical nephrectomy with psoas mass resection. Pathology confirmed recurrent clear-cell renal cell carcinoma with psoas muscle metastasis.

**Figure 3:**
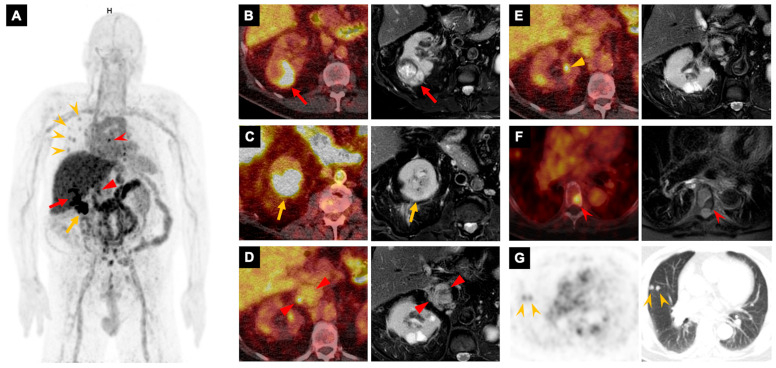
A 78-year-old man undergoing surveillance for Bosniak IIF left renal cysts was found to have an enlarging right interpolar renal mass. Shown are the ^89^Zr-girentuximab PET whole-body maximum intensity projection image (**A**), axial PET/CT fused images (**left** in (**B**–**F**)), axial T2-weighted fat-suppressed MRI (**right** in (**B**–**F**)), an axial PET image (**left** in (**G**)), and the corresponding lung-window CT image (**right** in (**G**)). The right interpolar mass demonstrates heterogeneously intense uptake (red arrows in (**A**,**B**)). Importantly, ^89^Zr-girentuximab PET reveals a second intensely avid right lower pole renal mass (yellow arrows in (**A**,**C**)) that is occult on MRI, where it exhibits only subtle T2 hypointensity. Additional foci of intense uptake are present within the expanded inferior vena cava (red triangles in (**A**,**D**)) and a right renal vein branch (yellow triangles in (**E**)), indicating tumor thrombus; the venous branch involvement is not conspicuous on MRI. A lesion in the T6 vertebral body (red arrowheads in (**A**,**F**)) and multiple pulmonary nodules (yellow arrowheads in (**A**,**G**)) also demonstrate uptake, consistent with metastatic disease. Biopsy of the right interpolar mass confirmed clear-cell renal cell carcinoma.

For renal lesions, oncocytomas (n = 8) demonstrated lower or comparable uptake relative to adjacent normal renal tissue (lesion-to-background ratio 0.4–1.3; median 0.5) (Figure 1, Supplementary Figures S1 and S2). In contrast, primary ccRCC lesions (n = 5) exhibited substantially higher uptake than the surrounding renal parenchyma (lesion-to-background ratio 4.4–17.3; median 10.5) (Figure 2 and Figure 3, Supplementary Figures S3 and S4).

For extrarenal extension, ^89^Zr-girentuximab PET/CT identified tumor thrombi within a renal vein branch (SUV_max_ 12.0) and the inferior vena cava (SUV_max_ 13.0), as well as a perinephric nodule (SUV_max_ 36.4). For metastatic disease, ^89^Zr-girentuximab PET/CT detected a psoas muscle lesion (SUV_max_ 103.0), a bone lesion (SUV_max_ 10.2), and multiple pulmonary nodules (n = 8; SUV_max_ 4.0–10.5, median 4.4). Benign lesions, including a perinephric nodule consistent with postoperative changes and a bone island, did not demonstrate uptake above the background of adjacent normal tissue (Table 1).

### 3.3. Comparison of Standardized Uptake Value Metrics

Clear-cell RCC lesions demonstrated significantly higher uptake than non-ccRCC lesions, reflected by higher lesion SUV_max_ [11.2 (4.0–103.0) vs. 5.4 (1.5–7.0), *p* = 0.018], lesion-to-background ratio [5.3 (3.4–147.1) vs. 0.7 (0.4–1.5), *p* < 0.001], lesion-to-liver ratio [1.6 (0.6–12.0) vs. 0.9 (0.2–1.3), *p* = 0.033], and lesion-to-blood-pool ratio [2.5 (0.9–18.4) vs. 1.2 (0.3–1.7), *p* = 0.014]. Similar findings were observed among biopsy-proven lesions (Table 2). Of note, the lesion-to-background ratio of ccRCC and non-ccRCC lesions showed no overlap.

Compared with primary ccRCC tumors, tumor thrombi and metastatic lesions showed greater variability in uptake intensity and overall lower uptake levels, as demonstrated by SUV_max_ [68.5 (17.3–91.7) vs. 6.5 (4.0–103.0), *p* = 0.010], lesion-to-liver ratio [9.4 (2.5–11.8) vs. 0.9 (0.6–12.0), *p* = 0.010], and lesion-to-blood pool ratio [10.2 (3.8–16.4) vs. 1.4 (0.9–18.4), *p* = 0.010]. The two groups were similarly conspicuous relative to adjacent normal tissue, with lesion-to-background ratios of 10.5 (4.4–17.3) for primary ccRCC tumors and 5.3 (3.4–147.1) for tumor thrombi and metastatic lesions, respectively. Notably, primary tumors were overall larger in size than tumor thrombi and metastatic lesions [54 (27–82) mm vs. 12 (8–103) mm, *p* = 0.008] (Table 3).

### 3.4. Impact on Clinical Management

Based on the survey of referring physicians, ^89^Zr-girentuximab PET/CT led to a change in clinical management in six of the seven patients (86%). In one patient (#3 in Table 1, Figure 2), the referring physician was uncertain at the time of the survey whether the imaging study altered management, as ^89^Zr-girentuximab PET/CT did not reveal additional sites of metastatic disease. All referring physicians either “strongly agreed” or “agreed” with the statement that “^89^Zr-girentuximab PET/CT improved patient care” in all seven cases.

^89^Zr-girentuximab PET/CT was particularly useful in complex clinical scenarios such as multiple renal masses, contraindication to biopsy or surgery, and indeterminate metastatic disease. For example, in a 76-year-old man with Birt–Hogg–Dubé syndrome (#1 in Table 1, Figure 1) who presented with multiple bilateral solid renal masses, biopsy of all lesions was not feasible. ^89^Zr-girentuximab PET/CT, together with biopsy confirmation of the dominant lesion, enabled a confident diagnosis of oncocytomatosis. Without PET/CT, the patient would likely have undergone partial nephrectomy or ablation; however, with PET/CT findings, he was appropriately managed with active imaging surveillance.

In Case #2 in Table 1, ^89^Zr-girentuximab PET/CT facilitated expedited care for a 63-year-old man with an incidental small right renal mass. The high ^89^Zr-girentuximab uptake suggested a high likelihood of ccRCC, prompting concurrent biopsy and microwave ablation in a single session. Pathologic analysis subsequently confirmed the diagnosis of ccRCC.

In Case #4 in Table 1, a 59-year-old man was evaluated for an incidental 7 cm left renal mass. Four months earlier, he had undergone coronary stent placement for acute myocardial infarction and remained on dual antiplatelet therapy, which could not be safely interrupted for at least 6 months and was ideally continued for 12 months. Holding antiplatelets for biopsy or surgery at that time carried a risk of major adverse cardiac events. To determine the level of urgency for intervention, ^89^Zr-girentuximab PET/CT was performed to distinguish aggressive histology, such as ccRCC, from benign or indolent tumors. The ^89^Zr-girentuximab PET/CT findings were most compatible with ccRCC, and the patient underwent left nephrectomy 6 months after stent placement.

In two additional cases (patients #5 and #6 in Table 1), patients presented with large incidental renal masses measuring >4 cm that, based on conventional clinical and imaging criteria, met indications for surgical resection because of concern for RCC [13]. ^89^Zr-girentuximab PET/CT findings were compatible with oncocytoma in both cases, allowing avoidance of substantial surgery with major renal function loss.

In Case #7 in Table 1 and Figure 3, ^89^Zr-girentuximab PET/CT provided critical incremental information in a 78-year-old man with an enlarging right interpolar renal mass and associated tumor thrombus involving the right renal vein and inferior vena cava on MRI. PET/CT detected a second right lower pole renal lesion with high ^89^Zr-girentuximab uptake that was occult on MRI. In addition, PET/CT identified a vertebral metastasis initially missed on MRI and confirmed the metastatic nature of multiple pulmonary nodules. These findings significantly changed the diagnostic impression and clinical approach.

## 4. Discussion

Indeterminate renal masses represent a significant unmet clinical need. Up to 30% of small renal masses resected by partial nephrectomy are ultimately benign, leading to unnecessary surgeries, costs, and morbidity [4,5]. Increasing evidence further indicates that small, indolent renal cell carcinoma histologies may be safely managed with active surveillance in selected patients [14,15,16]. ^89^Zr-girentuximab PET offers a novel noninvasive tool for characterizing indeterminate renal lesions. Our real-world single-center experience provides insight into how ^89^Zr-girentuximab PET/CT may be applied in clinical practice to address this unmet need and support decision-making in complex scenarios.

In our series, ^89^Zr-girentuximab PET/CT was straightforward to interpret when distinguishing ccRCC from non-ccRCC lesions. Visually, positive lesions typically exhibited uptake clearly above adjacent normal tissue, whereas negative lesions appeared photopenic or demonstrated uptake similar to background. We believe that PET positivity reflects on-target binding of the radiopharmaceutical to CAIX, whereas uptake similar to or lower than adjacent normal tissue in PET-negative lesions likely represents non-target-mediated uptake through variable mechanisms [17,18]. Compared with absolute SUV_max_, the lesion-to-background ratio may serve as a more robust discriminator, particularly for extrarenal lesions. Relative to primary ccRCC tumors, we observed greater variability in the degree of uptake in tumor thrombi and metastases, consistent with prior reports [19]. Although metastatic lesions may show higher uptake than the primary ccRCCs, small osseous and pulmonary metastases can also demonstrate low uptake. This observation may be partly due to their smaller size or variable levels of CAIX expression in such lesions.

Importantly, ^89^Zr-girentuximab PET/CT changed clinical management in most patients and was judged by referring physicians to improve patient care in all the recruited patients. We identified several clinical scenarios in which ^89^Zr-girentuximab PET/CT may be particularly helpful. (1) Characterization of multiple solid renal lesions when biopsy of all targets is not feasible or carries a significant risk: In such cases, if PET demonstrates uniform positive or negative uptake across all lesions, and the pathology of a biopsied dominant lesion is concordant with PET findings, the remaining lesions are highly likely to be of similar pathology. When ^89^Zr-girentuximab PET is negative, this may prevent unnecessary partial nephrectomy or additional biopsies. (2) Evaluation of the extent of local ccRCC: Our series demonstrates that ^89^Zr-girentuximab PET/CT can identify renal lesions that are occult on MRI and detect small branch tumor thrombi not appreciable on MRI. Additionally, ^89^Zr-girentuximab PET/CT may assist in distinguishing tumor thrombus from bland thrombus [11]. (3) Evaluation of suspected ccRCC metastases: ^89^Zr-girentuximab PET/CT may enhance the detection of metastatic sites that are challenging to identify on conventional imaging. Previous studies have shown that the detection rate of ^89^Zr-girentuximab PET/CT plus diagnostic CT exceeds that of ^18^F-FDG PET/CT plus CT, and of CT alone [19]. (4) Assessment of indeterminate extrarenal lesions: ^89^Zr-girentuximab PET can assist in resolving equivocal findings and differentiating metastases from benign lesions [11,20].

Filippi et al. proposed an algorithm for integrating ^89^Zr-girentuximab PET into clinical decision making [10]. Two aspects are particularly noteworthy: (1) For lesions that would traditionally require biopsy to guide management, ^89^Zr-girentuximab PET/CT may serve as an alternative, and PET-positive primary lesions may proceed directly to surgery without biopsy when clinically appropriate; (2) if the biopsy is nondiagnostic and the PET is negative, active surveillance is recommended rather than repeat biopsy or partial nephrectomy. Clinical follow-up and survey from referring providers in our study are concordant with both concepts. In addition, whether ^89^Zr-girentuximab PET can improve the confidence and accuracy of percutaneous needle biopsy results is an intriguing question.

Our study has several limitations. First, this study is a single-center experience with a small sample size, and its findings require confirmation in larger multicenter cohorts. We expect that broader real-world applications will further elucidate the clinical value of ^89^Zr-girentuximab PET/CT and potential challenges that may emerge. Second, the cohort is subject to selection bias inherent to an expanded access program, including referral bias for diagnostically challenging cases and clinician-driven enrollment. Third, histopathologic confirmation was not available for some of the evaluated lesions, and clinical diagnosis was used instead. These lesions were mainly non-dominant lesions or metastatic sites for which biopsy was either not feasible or not clinically indicated, as determined by the treating physicians. Despite these limitations, our findings provide valuable insight into how ^89^Zr-girentuximab PET/CT may be integrated into the clinical evaluation of IRMs and metastatic ccRCC.

## Figures and Tables

**Table 1:** Patient demographics, clinical characteristics, and lesion features.

**Patient No./** **Sex/Age (Year)**	**History**	**Site and Size of Lesions**	**Positivity on ^89^Zr-girentuximab PET**	**Lesion SUV_max_**	**Adjacent Normal Tissue SUV_mean_**	**Pathologic or Clinical Diagnosis**	**Patient Management Following ^89^Zr-girentuximab PET/CT**
1/M/76	Incidental multiple bilateral renal masses	Right kidney: 3 lesions (10–36 mm) Left kidney: 3 lesions (11–20 mm)	All negative	4.7–6.8	Kidney: 12.9	oncocytomas (biopsy of the largest right renal lesion)	Surveillance
2/M/63	Incidental right renal mass	Right kidney (27 mm)	Positive	68.5	Kidney: 15.5	ccRCC (P-Bx)	Microwave ablation at the time of biopsy
3/M/57	History of ccRCC s/p left partial nephrectomy 10 years ago. Recurrent left renal and psoas masses.	Left kidney (82 mm)	Positive	91.7	Kidney: 7.2	ccRCC (P-Ex)	Neoadjuvant systemic therapy followed by left radical nephrectomy with psoas deposit metastatectomy
		Left psoas muscle (26 mm)	Positive	103.0	Psoas muscle: 0.7	ccRCC (P-Ex)	
		Left anterior perinephric nodule (14 mm)	Positive	36.4	Kidney: 7.2	ccRCC (C)	
		Left inferior perinephric nodule (17 mm)	Negative	3.3	Kidney: 7.2	postsurgical changes (C)	
4/M/59	Incidental left renal mass	Left kidney (73 mm)	Positive	77.9	Kidney: 4.5	ccRCC (P-Ex)	Left nephrectomy
5/M/67	Incidental bilateral renal masses	Right kidney (61 mm)	Negative	7.0	Kidney: 5.3	oncocytoma (P-Bx)	Surveillance
		Left kidney (22 mm)	Negative	4.5	Kidney: 5.3	oncocytoma (C)	
6/M/76	Incidental left renal mass	Left kidney (52 mm)	Negative	5.5	Kidney: 4.6	oncocytoma (P-Bx)	Surveillance
7/M/78	Surveillance of Bosniak IIF left renal cysts. Enlarging right renal mass.	Right interpolar kidney (54 mm)	Positive	17.3	Kidney: 3.5	ccRCC (P-Bx)	Immunotherapy plus targeted therapy
		Right lower renal pole (57 mm)	Positive	36.9	Kidney: 3.5	ccRCC (C)	
		Suprarenal and perirenal IVC (103 mm)	Positive	13.0	Infrarenal IVC: 3.2	ccRCC tumor thrombus (C)	
		Right renal vein branch (18 mm)	Positive	12.0	Kidney: 3.5	ccRCC tumor thrombus (C)	
		Left kidney: 3 lesions (28–131 mm)	All negative	3.4–5.2	Kidney: 3.5	non-ccRCC renal neoplasms (C)	
		T6 vertebral body (18 mm)	Positive	10.2	Vertebra: 2.3	ccRCC bone metastasis (C)	
		Left acetabulum (16 mm)	Negative	1.5	Acetabulum: 1.6	bone island (C)	
		Lungs: 8 lesions (8–16 mm)	Positive	4.0–10.5	Lung: 0.8	ccRCC lung metastasis (C)	

P-Bx: pathologic diagnosis by biopsy; P-Ex: pathologic diagnosis by excision; C: Clinical diagnosis based on integrated findings from ^89^Zr-girentuximab PET/CT, conventional imaging, and clinical context. SUV: standardized uptake value. ccRCC: clear cell renal cell carcinoma. s/p: status post. IVC: inferior vena cava.

**Table 2:** Standardized uptake value metrics in ccRCC and non-ccRCC lesions.

	**All Lesions**			**Biopsy-Proven Lesions**		
	**ccRCC** **(n = 18)**	**Non-ccRCC** **(n = 14)**	***p*** **-Value**	**ccRCC** **(n = 5)**	**Oncocytoma** **(n = 3)**	***p*** **-Value**
Lesion SUV_max_	11.2 (4.0–103.0)	5.4 (1.5–7.0)	0.018	77.9 (17.3–103.0)	6.0 (5.5–7.0)	0.036
Lesion SUV_max_/adjacent normal tissue SUV_mean_	5.3 (3.4–147.1)	0.7 (0.4–1.5)	<0.001	12.7 (4.4–147.1)	1.2 (0.5–1.3)	0.036
Lesion SUV_max_/liver SUV_mean_	1.6 (0.6–12.0)	0.9 (0.2–1.3)	0.033	10.7 (2.5–12.0)	1.1 (0.9–1.1)	0.036
Lesion SUV_max_/blood pool SUV_mean_	2.5 (0.9–18.4)	1.2 (0.3–1.7)	0.014	16.2 (3.8–18.4)	1.2 (1.1–1.5)	0.036
Lesion largest diameter (mm)	17 (8–103)	21 (10–131)	0.37	54 (26–82)	52 (36–61)	1.00

Data are presented as median (range). ccRCC: clear cell renal cell carcinoma. SUV: standardized uptake value.

**Table 3:** Standardized uptake value metrics in primary tumors, tumor thrombi and metastatic lesions of ccRCC.

	**Primary Tumors of ccRCC (n = 5)**	**Tumor Thrombi or Metastatic Lesions of ccRCC (n = 13)**	***p*** **-Value**
Lesion SUV_max_	68.5 (17.3–91.7)	6.5 (4.0–103.0)	0.010
Lesion SUV_max_/adjacent normal tissue SUV_mean_	10.5 (4.4–17.3)	5.3 (3.4–147.1)	0.52
Lesion SUV_max_/liver SUV_mean_	9.4 (2.5–11.8)	0.9 (0.6–12.0)	0.010
Lesion SUV_max_/blood pool SUV_mean_	10.2 (3.8–16.4)	1.4 (0.9–18.4)	0.010
Lesion largest diameter (mm)	47 (27–82)	12 (8–103)	0.008

Data are presented as median (range). ccRCC: clear cell renal cell carcinoma. SUV: standardized uptake value.

## Data Availability

The data presented in this study are available from the corresponding author on reasonable request.

## Supplementary Materials

The following supporting information can be downloaded at: https://www.mdpi.com/article/10.3390/pharmaceutics18020258/s1, Figure S1: ^89^Zr-girentuximab PET/CT images of Patient #5; Figure S2: ^89^Zr-girentuximab PET/CT images of Patient #6; Figure S3: ^89^Zr-girentuximab PET/CT images of Patient #2; Figure S4: ^89^Zr-girentuximab PET/CT images of Patient #4; Table S1: ^89^Zr-girentuximab PET/CT acquisition protocol.

## Abbreviations

The following abbreviations are used in this manuscript:

IRM	Indeterminate renal mass
ccRCC	Clear-cell renal cell carcinoma
PET/CT	Positron emission tomography/computed tomography
EAP	Expanded access program
MRI	Magnetic resonance imaging
EOT	End of Treatment
SUV	Standardized uptake value
CAIX	Carbonic anhydrase IX

## References

[1] O’Connor S.D., Pickhardt P.J., Kim D.H., Oliva M.R., Silverman S.G. (2011). Incidental finding of renal masses at unenhanced CT: Prevalence and analysis of features for guiding management. AJR Am. J. Roentgenol..

[2] Roussel E., Capitanio U., Kutikov A., Oosterwijk E., Pedrosa I., Rowe S.P., Gorin M.A. (2022). Novel Imaging Methods for Renal Mass Characterization: A Collaborative Review. Eur. Urol..

[3] Pierorazio P.M., Hyams E.S., Mullins J.K., Allaf M.E. (2012). Active Surveillance for Small Renal Masses. Rev. Urol..

[4] van den Brink L., Debelle T., Gietelink L., Graafland N., Ruiter A., Bex A., Beerlage H.P., van Moorselaar R.J.A., Lagerveld B., Zondervan P. (2024). A National Study of the Rate of Benign Pathology After Partial Nephrectomy for T1 Renal Cell Carcinoma: Should We Be Satisfied?. Cancers.

[5] Kim J.H., Li S., Khandwala Y., Chung K.J., Park H.K., Chung B.I. (2019). Association of Prevalence of Benign Pathologic Findings After Partial Nephrectomy With Preoperative Imaging Patterns in the United States From 2007 to 2014. JAMA Surg..

[6] Chen K.T., Seimbille Y. (2022). New Developments in Carbonic Anhydrase IX-Targeted Fluorescence and Nuclear Imaging Agents. Int. J. Mol. Sci..

[7] Liao S.Y., Aurelio O.N., Jan K., Zavada J., Stanbridge E.J. (1997). Identification of the MN/CA9 protein as a reliable diagnostic biomarker of clear cell carcinoma of the kidney. Cancer Res..

[8] Bui M.H., Seligson D., Han K.R., Pantuck A.J., Dorey F.J., Huang Y., Horvath S., Leibovich B.C., Chopra S., Belldegrun A.S. (2003). Carbonic anhydrase IX is an independent predictor of survival in advanced renal clear cell carcinoma: Implications for prognosis and therapy. Clin. Cancer Res..

[9] Shuch B., Pantuck A.J., Bernhard J.C., Morris M.A., Master V., Scott A.M., van Praet C., Bailly C., Onal B., Aksoy T. (2024). [(89)Zr]Zr-girentuximab for PET-CT imaging of clear-cell renal cell carcinoma: A prospective, open-label, multicentre, phase 3 trial. Lancet Oncol..

[10] Filippi L., Urso L., D’Angelillo R.M., Evangelista L. (2025). Girentuximab imaging in renal cancer: Diamond in the rough or just ZIRCON?. Expert Rev. Anticancer. Ther..

[11] Hekman M.C.H., Rijpkema M., Aarntzen E.H., Mulder S.F., Langenhuijsen J.F., Oosterwijk E., Boerman O.C., Oyen W.J.G., Mulders P.F.A. (2018). Positron Emission Tomography/Computed Tomography with (89)Zr-girentuximab Can Aid in Diagnostic Dilemmas of Clear Cell Renal Cell Carcinoma Suspicion. Eur. Urol..

[12] 89Zr-DFO-girentuximab Expanded Access Program (EAP). https://clinicaltrials.gov/study/NCT06090331.

[13] National Comprehensive Cancer Network (2024). NCCN Clinical Practice Guidelines in Oncology: Kidney Cancer.

[14] Silverman S.G., Israel G.M., Trinh Q.-D. (2015). Incompletely Characterized Incidental Renal Masses: Emerging Data Support Conservative Management. Radiology.

[15] Monda S.M., Lui H.T., Pratsinis M.A., Chandrasekar T., Evans C.P., Dall’Era M.A. (2023). The Metastatic Risk of Renal Cell Carcinoma by Primary Tumor Size and Subtype. Eur. Urol. Open Sci..

[16] Finelli A., Cheung D.C., Al-Matar A., Evans A.J., Morash C.G., Pautler S.E., Siemens D.R., Tanguay S., Rendon R.A., Gleave M.E. (2020). Small Renal Mass Surveillance: Histology-specific Growth Rates in a Biopsy-characterized Cohort. Eur. Urol..

[17] Jauw Y.W., Menke-van der Houven van Oordt C.W., Hoekstra O.S., Hendrikse N.H., Vugts D.J., Zijlstra J.M., Huisamn H.C., van Dongen G.A. (2016). Immuno-positron emission tomography with zirconium-89-labeled monoclonal antibodies in oncology: What can we learn from initial clinical trials?. Front. Pharmacol..

[18] Huisman M.C., Pouw J.E., Golla S.S., Huglo D., Morschhauser F., Zijlstra J.M., Jauw Y.W.S., Boellaard R. (2025). 89Zr-mAb uptake interpretation requires the use of tissue to plasma ratios corrected for antibody catabolism. EJNMMI Res..

[19] Verhoeff S.R., van Es S.C., Boon E., van Helden E., Angus L., Elias S.G., Oosting S.F., Aarntzen E.H., Brouwers A.H., Kwee T.C. (2019). Lesion detection by [(89)Zr]Zr-DFO-girentuximab and [(18)F]FDG-PET/CT in patients with newly diagnosed metastatic renal cell carcinoma. Eur. J. Nucl. Med. Mol. Imaging.

[20] Verhoeff S.R., Oosting S.F., Elias S.G., van Es S.C., Gerritse S.L., Angus L., Heskamp S., Desar I.M.E., Menke-van der Houven van Oordt C.W., van der Veldt A.A.M. (2023). [89Zr]Zr-DFO-girentuximab and [18F]FDG PET/CT to Predict Watchful Waiting Duration in Patients with Metastatic Clear-cell Renal Cell Carcinoma. Clin. Cancer Res..

