# Supplementary material for: 89Zr-girentuximab PET/CT Enables Noninvasive Assessment of Indeterminate Renal Masses and Metastatic Clear-Cell Renal Cell Carcinoma
Source: Pharmaceutics. 2026 Feb 19;18(2):258. doi: 10.3390/pharmaceutics18020258 (PMC12944229; doi:10.3390/pharmaceutics18020258)
Supplement: Supplementary file 1 [file pharmaceutics-18-00258-s001.zip › Supplementary_Revised.pdf]

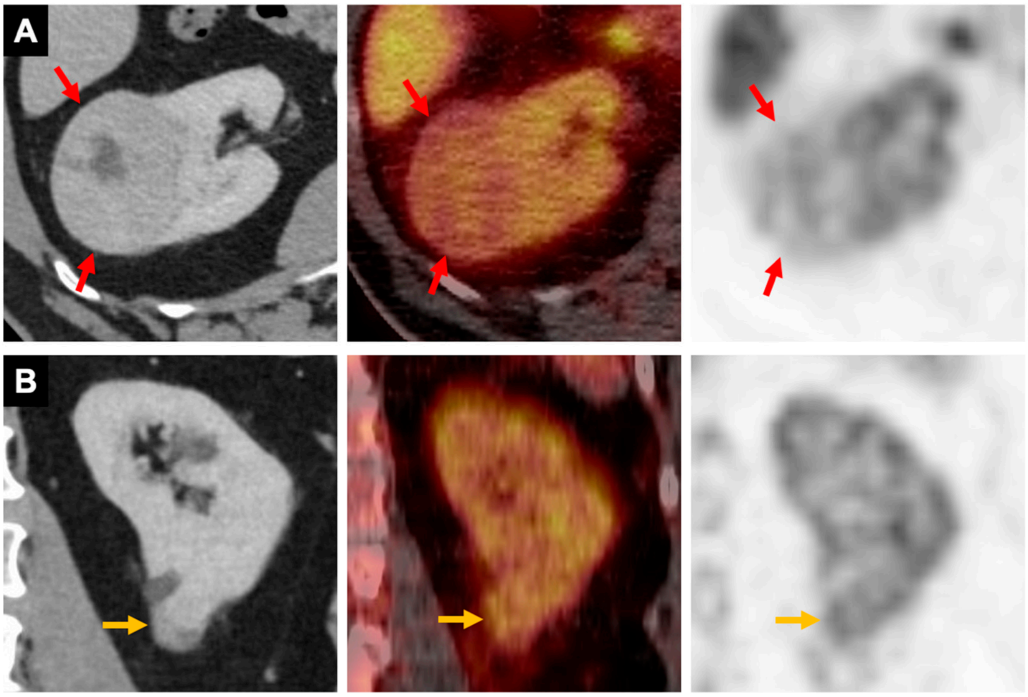

Supplementary Figure S1. A 67-year-old man (Patient #5 in Table 1) presented with incidental bilateral renal masses. Portal venous phase CT images (left), fused ^{89}Zr-girentuximab PET/CT images (middle), and PET images (right) demonstrate a 6-cm right renal mass (red arrows, row **A**) and a 2-cm left renal mass (yellow arrows, row **B**), both showing uptake similar to that of the adjacent normal renal parenchyma. Biopsy of the right renal mass revealed oncocytoma.

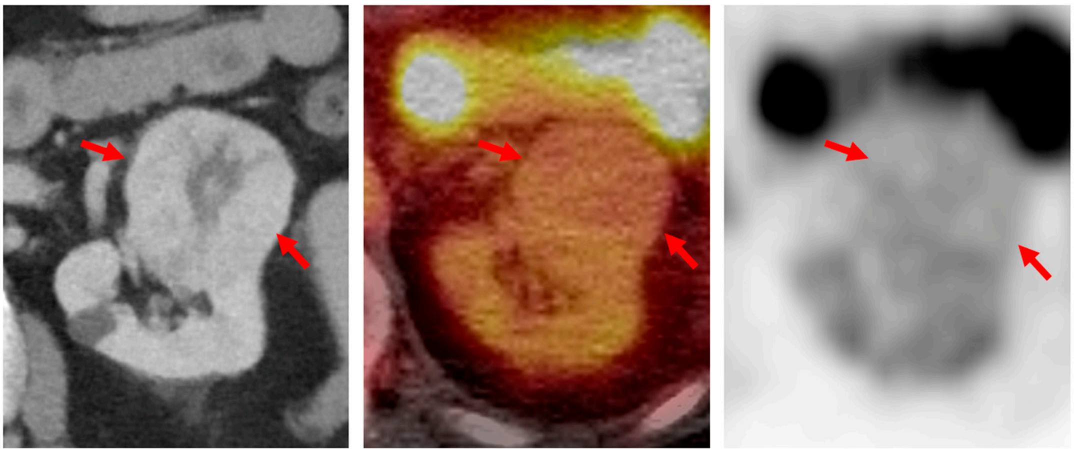

Supplementary Figure S2. A 76-year-old man (Patient #6 in Table 1) was evaluated for an incidental left renal mass. Axial portal venous phase CT image (left) demonstrates a 5-cm enhancing mass (arrows) in the left upper renal pole. The lesion exhibits uptake similar to that of the adjacent normal renal parenchyma on the fused ^{89}Zr-girentuximab PET/CT image (middle) and on the PET image alone (right). Subsequent biopsy confirmed the diagnosis of oncocytoma.

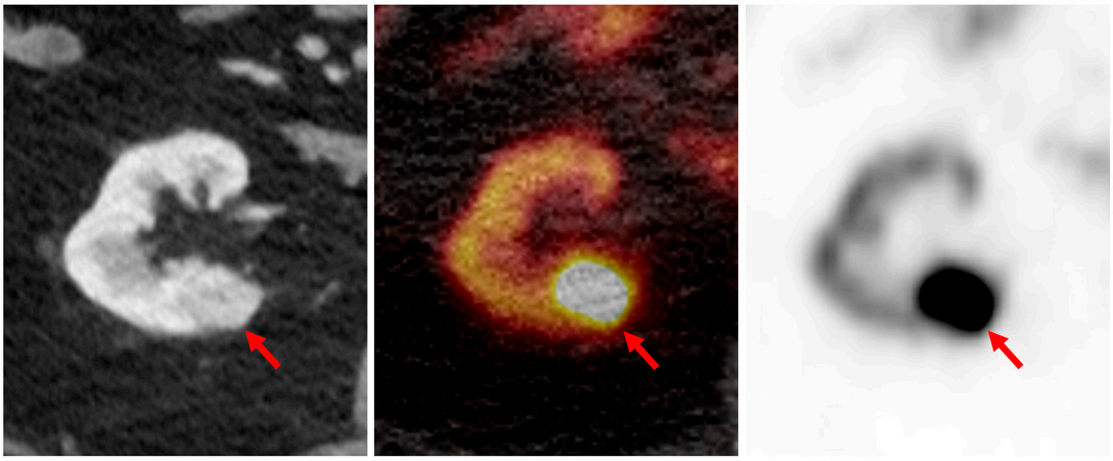

Supplementary Figure S3. A 63-year-old man (Patient #2 in Table 1) presented with an incidental right renal mass. Axial portal venous phase CT image (left) demonstrates an enhancing solid mass (arrows) in the right interpolar kidney. The lesion shows intense uptake on the fused ^{89}Zr-girentuximab PET/CT image (middle) and PET image (right). The patient subsequently underwent concurrent biopsy and microwave ablation, with pathology confirming clear cell renal cell carcinoma.

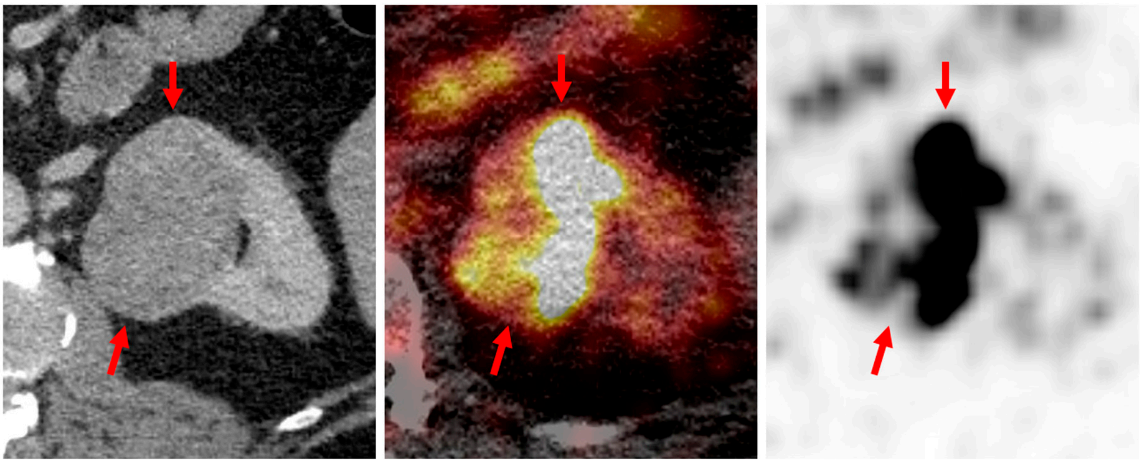

Supplementary Figure S4. A 59-year-old man (Patient #4 in Table 1) was evaluated for an incidental left renal mass. Axial portal venous phase CT image (left) demonstrates a 7-cm mass (arrows) in the left upper renal pole. The lesion exhibits heterogeneously intense uptake on the fused ^{89}Zr-girentuximab PET/CT image (middle) and PET image (right). The patient subsequently underwent left nephrectomy, with pathology confirming clear cell renal cell carcinoma.

Supplementary Table S1. PET/CT acquisition protocol

**Attenuation correction CT**			
Field of view (abdominal)	Top of liver through both kidneys	Effective mAs	30
Field of view (whole body)	Vertex to mid thighs	Extended FOV	780 mm
kV	120	Display FOV	500 mm
Slice	5 mm	Kernel	Br38
Acquisition	16 × 1.2 mm	Window	Abdomen
Rotation time	0.5 s	Reconstruction type	Axial
Pitch	1.5	Reconstruction increment	5.0 mm
Direction	Craniocaudal		
**Positron emission tomography**			
Field of view	To match attenuation CT	Zoom	1
Time/Bed (abdominal)	10-20 min/bed	Filter	Gaussian
Time/Bed (whole body)	5 min/bed	FWHM	6
Image size	220 mm	Scatter	Relative
Reconstruction	Iterative + Time of flight	Match CT slice	Yes
Iter/Subset	4/5	Volume Scaling	Yes

Scanner: Biograph Vision 600 PET/CT scanner (Siemens Healthineers, Knoxville, TN, USA).
