# Supplementary figures and images for: 89Zr-girentuximab PET/CT Enables Noninvasive Assessment of Indeterminate Renal Masses and Metastatic Clear-Cell Renal Cell Carcinoma
Source: Pharmaceutics. 2026 Feb 19;18(2):258. doi: 10.3390/pharmaceutics18020258 (PMC12944229; doi:10.3390/pharmaceutics18020258)

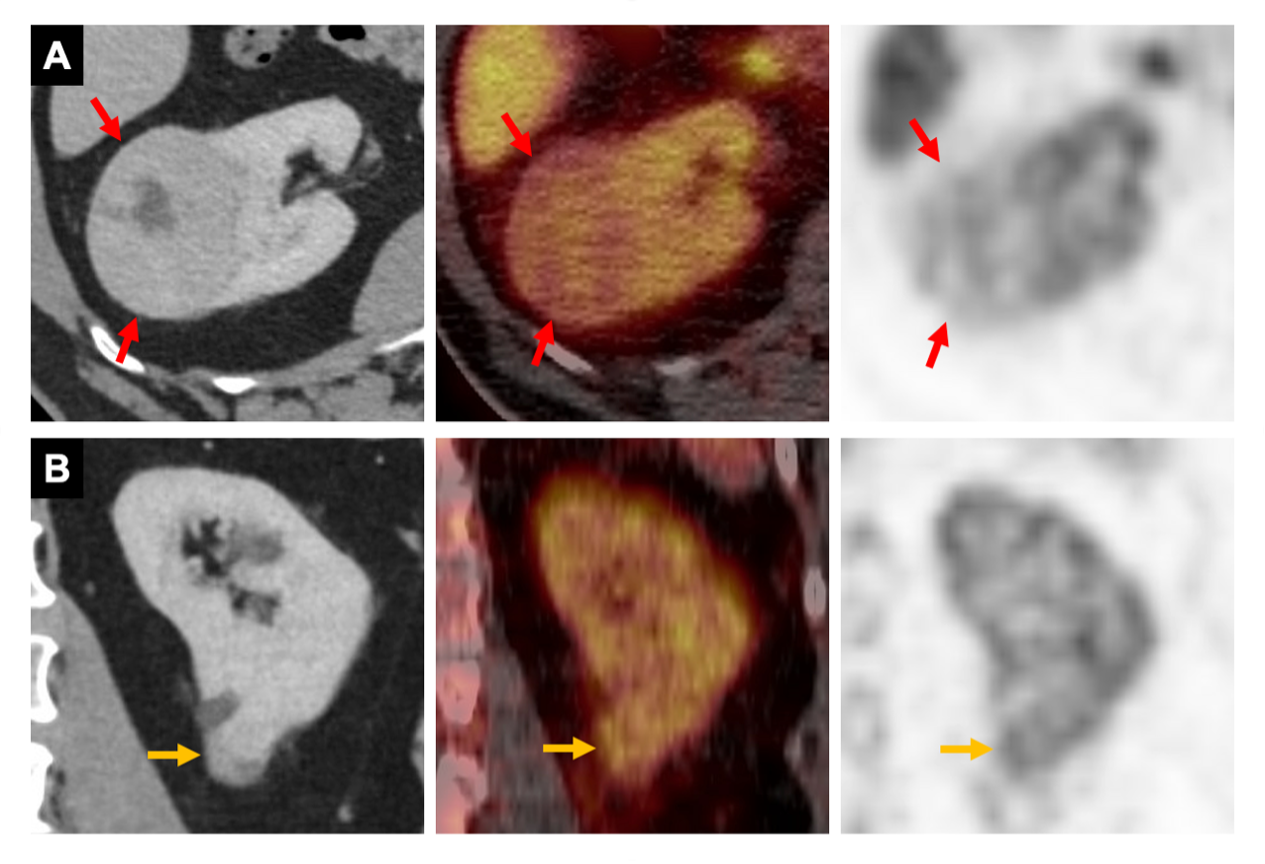

Supplement: Supplementary file 1 [file pharmaceutics-18-00258-s001.zip › Supplementary Figure 1.png]

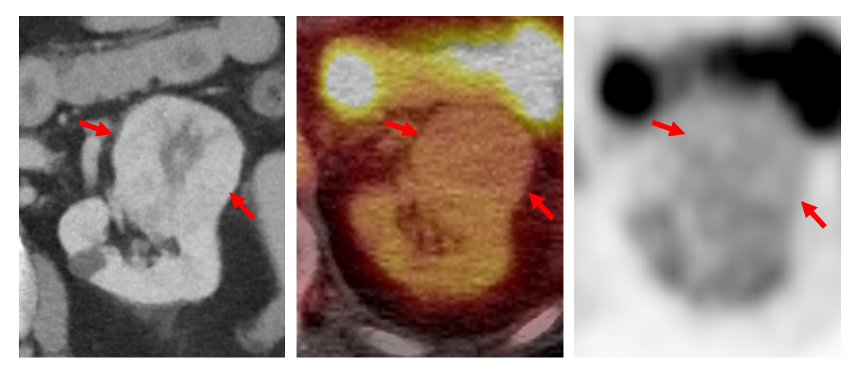

Supplement: Supplementary file 1 [file pharmaceutics-18-00258-s001.zip › Supplementary Figure 2.png]

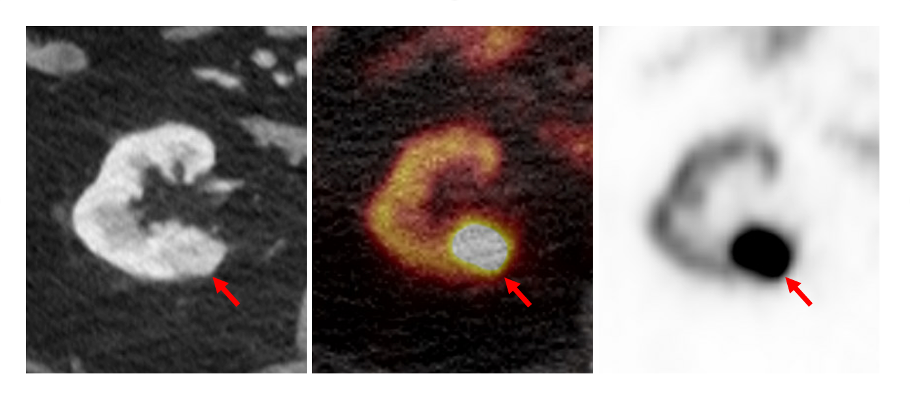

Supplement: Supplementary file 1 [file pharmaceutics-18-00258-s001.zip › Supplementary Figure 3.png]

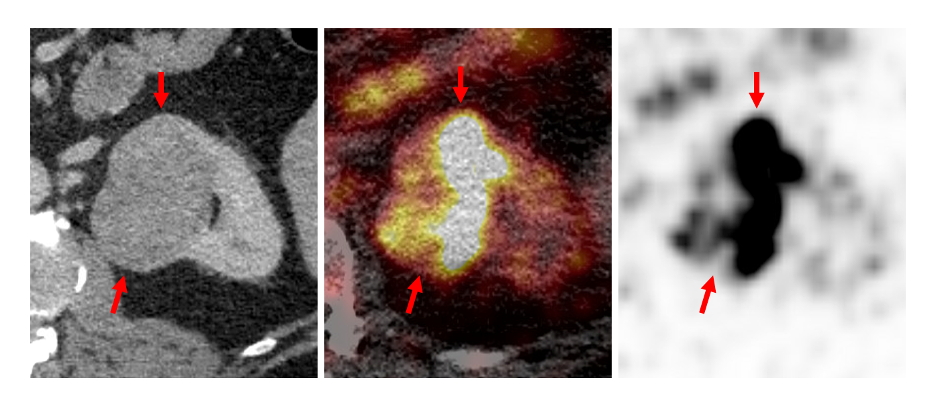

Supplement: Supplementary file 1 [file pharmaceutics-18-00258-s001.zip › Supplementary Figure 4.png]
